# Advanced Autonomous Underwater Vehicles Attitude Control with $L_{1}$ Backstepping Adaptive Control Strategy

**DOI:** 10.3390/s19224848

**Published:** 2019-11-07

**Authors:** Yuqian Liu, Jiaxing Che, Chengyu Cao

**Affiliations:** Department of Mechanical Engineering, University of Connecticut, Storrs CT 06269, USA; chengyu.cao@uconn.edu

**Keywords:** autonomous underwater vehicles, adaptive control, backstepping control, attitude control, stability analysis

## Abstract

This paper presents a novel attitude control design, which combines $L_{1}$ adaptive control and backstepping control together, for Autonomous Underwater Vehicles (AUVs) in a highly dynamic and uncertain environment. The Euler angle representation is adopted in this paper to represent the attitude propagation. Kinematics and dynamics of the attitude are in the strict feedback form, which leads the backstepping control strategy serving as the baseline controller. Moreover, by bringing fast and robust adaptation into the backstepping control architecture, our controller is capable of dealing with time-varying uncertainties from modeling and external disturbances in dynamics. This attitude controller is proposed for coupled pitch-yaw channels. For inevitable roll excursions, a Lyapunov function-based optimum linearization method is presented to analyze the stability of the roll angle in the operation region. Theoretical analysis and simulation results are given to demonstrate the feasibility of the developed control strategy.

## 1. Introduction

With increasing demands for undersea exploration and exploitation, researches in related fields have been given a boost, especially the study of autonomous underwater vehicles (AUVs), which is an undersea system containing its own power and controlling itself while accomplishing a pre-defined task [1]. A fully autonomous underwater robotics network for various missions with high performance is in great demand [2]. Thus, the design and control strategies of AUVs have received considerable attention by researchers.

The attitude control problem is one of the fundamental problems to facilitate the advancement of autonomous underwater networks. However, the attitude dynamics of AUVs have highly-coupled nonlinearity with uncertainties from modeling errors and time-varying external disturbances. Various methods have been proposed for the attitude control. Some work uses feedback controllers separately for each channel neglecting the coupling among angles, while some handle the problem directly by nonlinear control approaches, such as sliding-mode-based control [3,4,5,6], $H_{\infty }$ tools [7,8] and others.

In this paper, based on good study of the nonlinear AUV model presented in [9], which is driven by four water pumps, a precise attitude controller with the combination of backstepping control and $L_{1}$ adaptive control is proposed for the yaw and pitch channels. The backstepping approach provides a recursive method for stabilizing the origin of a system in strict-feedback form [10]. The kinematics and dynamics of the attitude problem for mobile system with 6-DOF are in strict feedback form, which is a good application of backstepping control strategy [11]. In order to handle large time-varying uncertainties in dynamics, which are from modeling, complicated hydrodynamics, and external disturbances, we bring the $L_{1}$ adaptive control theory into the backstepping control architecture. The key feature of $L_{1}$ adaptive control is guaranteed robustness in the presence of fast adaptation [12]. It is a modification of model reference adaptive control (MRAC) and was initially motivated by aerospace applications. With the combination of high gain and low-pass filter, it guarantees fast adaptation and satisfactory transient response. Theoretical results, analysis details and extensions can be found in [12,13,14,15,16,17,18]. Thus, we chose the $L_{1}$ backstepping adaptive control architecture to achieve robust attitude control of AUVs.

Moreover, due to physical design, the AUV could only provide sufficient moments on the pitch and yaw channels. The transient response analysis for pitch and yaw control is well studied with inspiration form the works in [11,19]. Unwanted roll excursions are inevitable and dynamically coupled into yaw and pitch motion [8]. The roll motion can be ambiguous without active roll stabilization, especially for smaller AUVs which have a relatively small stabilization moment due to limited vertical distance from the center of gravity to the center of buoyancy. Thus, we introduce a Lyapunov function-based optimum method to analyze the stability of roll angle in the operation region. Simulation results are presented to study and improve the design.

The rest of paper is organized as follows: Section 2 briefly introduces the advanced AUV design presented in [9]. Section 3 presents the nonlinear model and the attitude control. Section 4 introduces attitude controller design based on $L_{1}$ backstepping control only considering uncertainties in dynamics. In Section 5, we provide the proof of guaranteed transient response analysis for the coupled pitch-yaw channels. The stability of the roll angle is also discussed here. The simulation results are presented in Section 6. Section 7 gives the conclusion and future work.

## 2. AUV Mechanical Design

**Figure 1:**
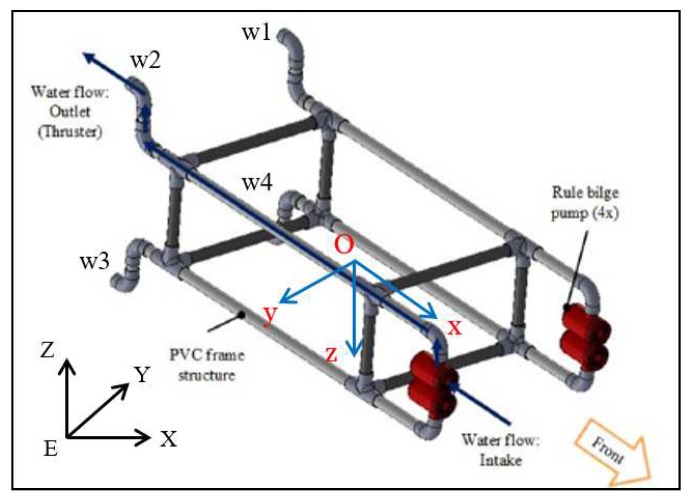
Mechanical design and propulsion system.

The attitude controller design is based on a full-scale underwater platform depicted in Figure 1, which utilizes a thrust propulsion system powered by four low-cost submersible pumps forming four water-jet thrusters with reducing end nozzles [9]. This provides an optimal thrust to flow rate ratio. By differentiating the power combinations of four motors, the force and moments needed for the propulsion and maneuvering are generated. Figure 1 shows the working mode of AUV, in which the force vector points to the back and two degree of freedoms (DOFs) of the moment to manipulate the pitch and yaw angles. The manufacturing cost is low compared to the deep-water design which would require more control surfaces.

This AUV is aligned to be neutrally buoyant, which means the gravitational force and buoyant force are equal to each other. It is also aligned to be naturally stable, which means two of the three Euler angles, ϕ and θ, are close to zero when it is at rest. The body coordinate frame *O* is attached to the center of mass of the AUV. The *x* axis points to the head and y axis goes to the right of the AUV. More modeling details are presented in Appendix A.

## 3. Problem Formulation and Control Objective

### 3.1. Kinematics and Dynamics of Attitude

The Euler angle representation is used here to present the attitude of the AUV. Table 1 gives some of the definitions and notation in this paper.

A standard notation for attitude propagation equations and attitude dynamics is adopted here,

(1) $$\begin{array}{c} \begin{array}{c} \left[\begin{array}{c} \dot{\Omega }\\ J\dot{w} \end{array}\right]=\left[\begin{array}{c} \Psi (\Omega )w\\ \eta^{b}-w\times Jw \end{array}\right] \end{array}\;. \end{array}$$

The first line of Equation (1) describes the kinematics of attitude angles, where $\Omega ={\left[\begin{array}{ccc} \phi & \theta & \psi \end{array}\right]}^{T}$ and $w={\left[\begin{array}{ccc} w_{x} & w_{y} & w_{z} \end{array}\right]}^{T}$ are state vectors of attitude angles and angle rates expressed in the body frame. ϕ, θ and ψ denote roll, pitch and yaw angle, while $w_{x}$, $w_{y}$ and $w_{z}$ are angle rates with respect to *x*, *y* and *z* axes of body frame, and

(2) $$\begin{array}{ccc} \Psi (\Omega ) & = & \left[\begin{array}{ccc} 1 & s\phi t\theta & c\phi t\theta \\ 0 & c\phi & -s\phi \\ 0 & s\phi /c\theta & c\phi /c\theta \end{array}\right]\;. \end{array}$$

The second line of Equation (1) describes the dynamics of attitude angles, where the diagonal matrix $J\in R^{3}$ is moment of inertia of the AUV, and $\eta^{b}\in R^{3}$ is the overall moment applied on the AUV expressed in body frame, which consists of buoyancy–gravity stabilization moment $\tau_{G}$, control input η and fluid dynamic moment $\tau_{D}$, shown as follows,

(3) $$\begin{array}{ccc} \eta^{b} & = & \tau_{G}+\eta +\tau_{D}\;. \end{array}$$

As mentioned before, this could offer two DOFs of moment to manipulate the pitch and yaw angle. Thus, the control signal is $\eta ={\left[0\;\;\;\eta_{y}\;\;\;\eta_{z}\right]}^{T}$.

Equation (1) summarizes the overall attitude model of the AUV. The modeling details could be found in [9].

This paper only considers the uncertainties in dynamics. Thus, compared to Equation (1), the attitude dynamics can be represented as follows,

(4) $$\begin{array}{c} \dot{w}=\bar{H}\left(w\right)+{\bar{H}}_{\delta }\left(w\right)+\left(K+K_{\delta }\right)\eta^{b}+K\sigma_{out}, \end{array}$$

where $\bar{H}\left(w\right)=-J^{-1}\left(w\times Jw\right)$ and $K=J^{-1}$. The uncertainties, ${\bar{H}}_{\delta }\left(w\right)$ and $K_{\delta }$, are due to the imprecise knowledge of *J*. $\sigma_{out}$ represents disturbance from environment. This dynamic equation could be

(5) $$\begin{array}{c} \dot{w}=\bar{H}\left(w\right)+K\left(K^{-1}{\bar{H}}_{\delta }\left(w\right)+K^{-1}K_{\delta }\eta^{b}+\eta^{b}+\sigma_{out}\right)\;. \end{array}$$

Expanding the second $\eta^{b}$ in (5), let $H\left(w\right)=\bar{H}\left(w\right)+\tau_{G}+\tau_{D}$, and defining $\sigma =K^{-1}K_{\delta }\eta^{b}+K^{-1}{\bar{H}}_{\delta }\left(w\right)+\sigma_{out}$ to represent the overall uncertainties, we obtain

(6) $$\begin{array}{c} \dot{w}=H\left(w\right)+K\left(\eta +\sigma \right). \end{array}$$

### 3.2. The Trimmed Model for Pitch and Yaw Dynamics

In this section, the original model in Section 3.1 is trimmed for controller design and performance analysis.

Define

(7) $$\begin{array}{c} I_{s}=\left[\begin{array}{ccc} 0 & 1 & 0\\ 0 & 0 & 1 \end{array}\right]; \end{array}$$

the kinematics Euler angle equations of the pitch and yaw channels are

(8) $$\begin{array}{ccc} \left[\begin{array}{c} \dot{\theta }\\ \dot{\psi } \end{array}\right] & = & \left[\begin{array}{cc} c\phi & -s\phi \\ s\phi /c\theta & c\phi /c\theta \end{array}\right]\left[\begin{array}{c} w_{y}\\ w_{z} \end{array}\right]\;,\\ \left[\begin{array}{c} {\dot{w}}_{y}\\ {\dot{w}}_{z} \end{array}\right] & = & I_{s}\dot{w}=I_{s}H\left(w\right)+I_{s}K\left(\eta +\sigma \right). \end{array}$$

Define $\eta_{s}=\left[\begin{array}{c} \tau_{y}\\ \tau_{z} \end{array}\right]$ as the control signal. To further simply Equation (8), we introduce the following definitions.

$$\begin{array}{c} \begin{array}{cccccc} w_{s} & =I_{s}w=\left[\begin{array}{c} w_{y}\\ w_{z} \end{array}\right], & \;\;\Omega_{s} & =I_{s}\Omega =\left[\begin{array}{c} \theta \\ \psi \end{array}\right], & \;\;\sigma_{s} & =I_{s}\sigma ,\\ K_{s} & =I_{s}KI_{s}^{T}, & \;\;J_{s} & =I_{s}JI_{s}^{T}, \end{array}\\ \begin{array}{cc} \Psi_{s}\left(\Omega \right) & =I_{s}\Psi \left(\Omega \right)I_{s}^{T}=\left[\begin{array}{cc} c\phi & -s\phi \\ s\phi /c\theta & c\phi /c\theta \end{array}\right],\\ H_{s}\left(w\right) & =I_{s}H\left(w\right)=\left[\begin{array}{c} -J_{22}^{-1}w_{x}w_{z}\left(J_{11}-J_{33}\right)\\ -J_{33}^{-1}w_{x}w_{y}\left(J_{22}-J_{11}\right) \end{array}\right], \end{array} \end{array}$$

where $J_{11}$, $J_{22}$ and $J_{33}$ are the diagonal elements of *J*.

Thus, Equation (8) is

(9) $$\begin{array}{ccc} {\dot{\Omega }}_{s} & = & \Psi_{s}\left(\Omega \right)w_{s}\\ {\dot{w}}_{s} & = & H_{s}\left(w\right)+K_{s}\left(\eta_{s}+\sigma_{s}\right). \end{array}$$

All the uncertainties have been added together into $\sigma_{s}\in R$, which is subject to the following assumptions:

**Assumption** **1.** *There exist constants L>0 and $L_{0}>0$ such that the following inequalities hold uniformly in t≥0, $\forall w_{1},w_{2}\in R{}^{3}$ and $\forall w_{s_{1}},w_{s_{2}}\in R{}^{2}$:*

(10) $$\begin{array}{ccc} ∥\sigma \left(t,w_{1}\right)-\sigma \left(t,w_{2}\right){∥}_{\infty } & \leq & L||w_{1}-w_{2}{\left|\right|}_{\infty }+L_{0}\;\; \end{array}$$

**Assumption** **2.** *There exist constants $L_{1}>0$, $L_{2}>0$ and $L_{3}>0$ such that the following inequalities hold uniformly in t≥0:*

(11) $$\begin{array}{ccc} {∥\dot{\sigma }\left(t\right)∥}_{\infty } & \leq & L_{1}∥\dot{w}{\left(t\right)∥}_{\infty }+L_{2}{∥w\left(t\right)∥}_{\infty }+L_{3}\; \end{array}$$

**Assumption** **3.** *The desired command $\Omega_{d},{\dot{\Omega }}_{d}$ and ${\ddot{\Omega }}_{d}\in R^{2}$ are bounded. Ω and ω exist in $\bar{\Omega }$ and $\bar{w}$, which are compact subsets of $R^{3}$, where Ψ(Ω) and H(w) are continuous and bounded, and so are $\Omega_{s}$ and $\omega_{s}$*.

### 3.3. Control Objective

The control objective is to design an adaptive attitude controller, which could let attitude angles to track the desired commands $\Omega_{d}$. In this paper, the goal is to let the pitch and yaw angles, $\Omega_{s}$, always track the desired values, $\Omega_{d}$. Meanwhile, the roll angle is self-stable. The self-stability of the roll angle will be proved in the analysis part.

## 4. Controller Design

### 4.1. State Predictor

The state predictor is defined as follows,

(12) $$\begin{array}{c} \begin{array}{c} {\dot{\hat{w}}}_{s}\overset{\Delta }{\;=}H_{s}\left(w\right)+K_{s}\left(\eta_{s}+{\hat{\sigma }}_{s}\right)+A_{m}e, \end{array} \end{array}$$

where $e\overset{\Delta }{\;=}{\hat{w}}_{s}-w_{s}$ is the prediction error, and $A_{m}$ is a Hurwitz matrix, which defines the desired convergence of *e*. Using ${\tilde{\sigma }}_{s}={\hat{\sigma }}_{s}-\sigma_{s}$, the prediction error is,

(13) $$\begin{array}{c} \dot{e}=A_{m}e+K\left({\hat{\sigma }}_{s}-\sigma_{s}\right)\;.\; \end{array}$$

The Laplace transform of it is,

(14) $$\begin{array}{c} e\left(s\right)={\left(sI-A_{m}\right)}^{-1}K{\tilde{\sigma }}_{s}\;.\; \end{array}$$

### 4.2. Adaptive Law

Setting the sampling time of the adaptation law by $T_{s}$, and the prediction error by *e*, a piecewise constant adaptation law is given by

(15) $$\begin{array}{c} \hat{\sigma }\left(iT_{s}\right)=J_{s}\phi \left(T_{s}\right)u\left(iT_{s}\right),\;\;\forall t\in \left[iT_{s},\left(i+1\right)T_{s}\right], \end{array}$$

where $\phi \left(T_{s}\right)=A_{m}{\left(I-exp\left(A_{m}T_{s}\right)\right)}^{-1}$ and $u\left(iT_{s}\right)=exp\left(A_{m}T_{s}\right)e\left(iT_{s}\right)$ for all i=1,2,3,....

### 4.3. $L_{1}$ Backstepping Euler Angle Controller

Define $\eta_{s}={\left[\eta_{y}\;\;\;\eta_{z}\right]}^{T}$, and use $\eta_{b}\in R^{2}$ and $\eta_{a}\in R^{2}$ to represent control laws coming from the backstepping loop and $L_{1}$ adaptive loop respectively. Thus,

(16) $$\begin{array}{c} \eta_{s}=\eta_{b}+\eta_{a}, \end{array}$$

where

(17) $$\begin{array}{ccc} \eta_{b} & = & J_{s}\left[A_{m2}\left(w_{s}-w_{d}\right)-H_{s}\left(w\right)-\Psi_{s}^{T}\left(\Omega \right)\left(\Omega_{s}-\Omega_{d}\right)\right] \end{array}$$

(18) $$\begin{array}{ccc} \eta_{a} & = & -C\left(s\right)\left({\hat{\sigma }}_{s}-\eta_{{\dot{w}}_{d}}\right) \end{array}$$

(19) $$\begin{array}{ccc} \eta_{{\dot{w}}_{d}} & = & Jsw_{d} \end{array}$$

(20) $$\begin{array}{ccc} w_{s_{d}} & = & \Psi^{-1}\left(A_{m1}\left(\Omega_{s}-\Omega_{d}\right)+{\dot{\Omega }}_{d}\right) \end{array}$$

$A_{m1},A_{m2}$ are diagonal Hurwitz matrices. $K_{1}$ is a positive gain and $D_{1}\left(s\right)$ is a strictly proper transfer function, the value of which ensure $C\left(s\right)=\frac{K_{1}D_{1}}{1+K_{1}D_{1}}$ has unit DC gain.

For the entire system in (1), the overall control law would be

(21) $$\begin{array}{c} \eta =\left[\begin{array}{c} 0\\ \eta_{s} \end{array}\right]\;. \end{array}$$

## 5. Analysis

The performance analysis of this paper has two parts, namely, self-stability analysis in the roll angle channel and response performance analysis in the pitch-yaw angle channels. The roll angle channel relies on the self stabilization mechanism of itself with assumptions of bounded states in the pitch-yaw channels. Then, the pitch-yaw angle channels stability based on the bounded states in the roll channel is analyzed. The overall stability of the system will be discussed at the end.

### 5.1. Roll Angle Channel Self-Stability Analysis

Expanding Equation (6), the dynamics of the roll angle is described by the following equations,

(22) $$\begin{array}{cc} \dot{\phi } & =w_{x}+\left[\begin{array}{cc} s\theta t\theta & c\phi t\theta \end{array}\right]\left[\begin{array}{c} w_{y}\\ w_{z} \end{array}\right] \end{array}$$

(23) $$\begin{array}{cc} {\dot{w}}_{x} & =-J_{11}^{-1}G_{W}d\sin\phi -\frac{1}{2}C_{x}sign\left(w_{x}\right)w_{x}^{2}-J_{11}^{-1}w_{y}w_{z}\left(J_{33}-J_{22}\right)+\sigma_{1}, \end{array}$$

where $\sigma_{1}=\left[\begin{array}{ccc} 1 & 0 & 0 \end{array}\right]\sigma$, representing the overall uncertainty in the roll channel. The first two terms in ${\dot{w}}_{x}$ come from $\tau_{G}$ and $\tau_{D}$, where *d* is the distance from the center of gravity and the center of buoyancy, and $C_{x}$ is the damping coefficient.

Define

(24) $$\begin{array}{ccc} d_{1} & = & \left[\begin{array}{cc} s\theta t\theta & c\phi t\theta \end{array}\right]\left[\begin{array}{c} w_{y}\\ w_{z} \end{array}\right] \end{array}$$

(25) $$\begin{array}{ccc} d_{2} & = & J_{11}^{-1}w_{y}w_{z}\left(J_{33}-J_{22}\right)+\sigma_{1}, \end{array}$$

Equations (22) and (23) can be written as follows,

(26) $$\begin{array}{ccc} \dot{\phi } & = & w_{x}+d_{1} \end{array}$$

(27) $$\begin{array}{ccc} {\dot{w}}_{x} & = & -J_{11}^{-1}G_{W}d\sin\phi -\frac{1}{2}C_{x}sign\left(w_{x}\right)w_{x}^{2}+d_{2}\;. \end{array}$$

Consider the linearization model,

(28) $$\begin{array}{c} \dot{x}=Ax+d_{roll}, \end{array}$$

where $x=\left[\begin{array}{c} \phi \\ w_{x} \end{array}\right]$ is the state vector, and $A=\left[\begin{array}{cc} 0 & 1\\ -g_{1}g_{2} & -\frac{1}{2}C_{x}g_{3} \end{array}\right]$ is a Hurwitz matrix. $g_{1}=J_{11}^{-1}G_{W}d$ is a positive constant. $g_{2}$ and $g_{3}$ are positive linearization coefficients. $d_{roll}\in R^{2}$ is the uncertainty that

(29) $$\begin{array}{c} d_{roll}=d+d_{m}, \end{array}$$

where $d={\left[\begin{array}{cc} d_{1} & d_{2} \end{array}\right]}^{T}$ is the uncertainty from the other two channels, and $d_{m}={\left[\begin{array}{cc} 0 & d_{3} \end{array}\right]}^{T}$ represents the difference between the linearization model and the original model of *x*. $d_{1}$, $d_{2}$ are defined in (24) and (25), while $d_{3}$ is defined as follows.

$$\begin{array}{c} d_{3}=g_{1}\left(g_{2}\phi -\sin\phi \right)+\frac{1}{2}C_{x}\left(g_{3}w_{x}-sign\left(w_{x}\right)w_{x}^{2}\right)\;.\; \end{array}$$

Since *A* is a Hurwitz matrix, there exist matrices *P* and *Q*, which are positive definite. Consider the Lyapunov function candidate:

(30) $$\begin{array}{c} V_{roll}\left(x\left(t\right)\right)=x^{T}\left(t\right)Px\left(t\right)\;.\; \end{array}$$

The derivative of $V_{roll}$ is

(31) $$\begin{array}{ccc} {\dot{V}}_{roll}\left(x\left(t\right)\right) & = & -x^{T}\left(t\right)Qx\left(t\right)+2x^{T}\left(t\right)Pd_{roll}\left(t\right)\\ & \leq & -x^{T}\left(t\right)Qx\left(t\right)+2x^{T}\left(t\right)Pd_{m}\left(t\right)+2\lambda_{\max}\left(P\right)∥x(t)∥∥d∥\;.\; \end{array}$$

$d_{1}$ and $d_{2}$ are continuous and bounded in the compact sets $\bar{\Omega }$ and $\bar{\omega }$, such that

(32) $$\begin{array}{ccc} ∥d∥ & \leq & ∥d_{1}∥+∥d_{2}∥\\ & = & b_{d1}+b_{d2}\;.\; \end{array}$$

Thus, (31) could be written as

(33) $$\begin{array}{c} \dot{V}{\left(t\right)}_{roll}\leq -x^{T}\left(t\right)Qx\left(t\right)+2x^{T}\left(t\right)Pd_{m}\left(t\right)+2\lambda_{\max}\left(P\right)∥x(t)∥\left(b_{d1}+b_{d2}\right), \end{array}$$

which shows the boundary of ${\dot{V}}_{roll}$ is effected by the value of the linearization mismatch error $d_{3}$ in $d_{m}$. With the optimum linearization, proper coefficients, $g_{2}$ and $g_{3}$, could give the minimum $d_{3}$.

**Lemma** **1.** *For the $V_{roll}\left(x\left(t\right)\right)$ and ${\dot{V}}_{roll}\left(x\left(t\right)\right)$ defined in (30) and (31) if there exist $b_{d1}$, $b_{d2}$, $b_{x}$ such that*

(34) $$\begin{array}{cc} i & \;\;∥{d_{1}}_{t_{1}}∥\leq b_{d1}\;∥{d_{2}}_{t_{1}}∥\leq b_{d2}\;. \end{array}$$

(35) $$\begin{array}{cc} ii & \;\;{\dot{V}}_{roll}\left(x\right)\leq 0,\;\forall x\in \left\{x|\;V_{roll}\left(x\right)=b_{x}^{2}\lambda_{\min}\left(P\right)\right\}\;. \end{array}$$

*Then the roll channel is bounded and*

(36) $$\begin{array}{c} {∥x_{t_{1}}∥}_{L\infty }\leq b_{x}\;. \end{array}$$

*where $t_{1}>0$ is a dummy variable*.

**Proof of Lemma** **1.** Recalling the Lyapunov function in (30), first we prove that

(37) $$\begin{array}{c} ∥V_{roll_{t_{1}}}∥\leq {b_{x}}^{2}\lambda_{\min}\left(P\right) \end{array}$$

by contradiction method as follows. Assume the opposite of Equation (37) is true, then there exists some time $t\in [0,t_{1}]$ that

(38) $$\begin{array}{c} V_{roll}\left(t\right)>{b_{x}}^{2}\lambda_{\min}\left(P\right)\;. \end{array}$$

Since $V_{roll}\left(0\right)<b_{x}\lambda_{\min}\left(P\right)$, and V(t) is continuous, there exists a time $t^{\prime }\in \left[0,t_{1}\right]$, such that

(39) $$\begin{array}{c} V_{roll}\left(t^{\prime }\right)={b_{x}}^{2}\lambda_{\min}\left(P\right), \end{array}$$

while

(40) $$\begin{array}{c} V_{roll}\left(t_{-}^{\prime }\right)<{b_{x}}^{2}\lambda_{\min}\left(P\right)\;.\; \end{array}$$

Equations (39) and (40) imply that $\dot{V}\left(t^{\prime }\right)>0$, which is clearly in contradiction with *Condition ii* in (35). Thus, Equation (37) is true, holding for all $t\in [0,t_{1}]$,

$$\begin{array}{c} V_{roll}\left(t\right)\leq {b_{x}}^{2}\lambda_{\min}\left(P\right)\;.\; \end{array}$$

Since $\lambda_{\min}\left(P\right){∥x∥}^{2}\leq x^{T}\left(t\right)Px\left(t\right)=V\left(t\right)$, then $\forall \;t\in [0,t_{1}]$

(41) $$\begin{array}{ccc} {∥x∥}^{2} & \leq & \frac{b_{x}^{2}\lambda_{\min}\left(P\right)}{\lambda_{\min}\left(P\right)},\\ & \leq & b_{x}^{2}\;, \end{array}$$

which leads to (36). □

**Figure 2:**
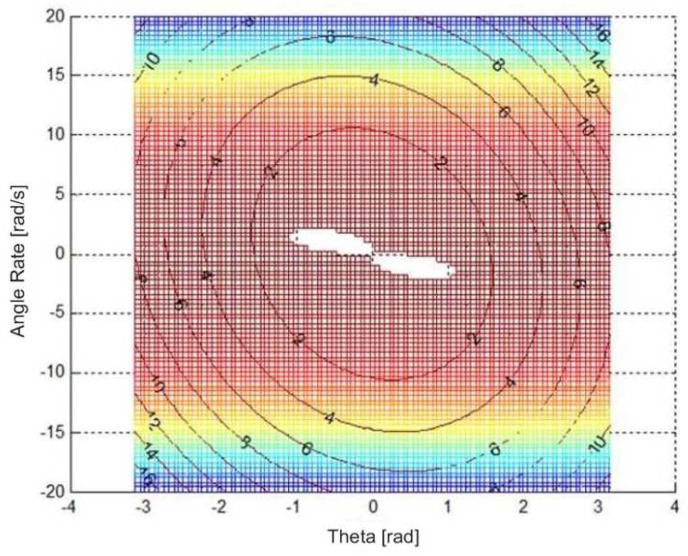
V(x) and $\dot{V}\left(x\right)$ in the roll channel.

**Remark** **1.** *For a given pair of $g_{2}$ and $g_{3}$, with an appropriate choice of P and Q, the Lyapunov function and the derivative of it are specified, which are shown in Figure 2. The blank region in the center indicts where $\dot{V}\left(x\right)>0$, while the colorful region shows where $\dot{V}\left(x\right)<0$. The contours represent $V\left(x\right)=V_{c}$, where $V_{c}\in R$. Figure 2 shows a bunch of Lyapunov contours with different values of $V_{c}$. Any contours within the colorful region satisfy* Condition ii *in Lemma 1*.

**Optimum Linearization.** In what follows, we introduce an optimum procedure to find $g_{2}$ and g3, such that $\dot{V}>0$ is a closed region, which means contours $V\left(x\right)=V_{c}$ exist, and find the minimum value of $V_{c}$, denoted as $V_{c_{min}}$.

1. For a given pair of $\left(g_{2},g_{3}\right)$, with the specified *P* and *Q*, we have the objective function $L\left(x\right)=-\dot{V}\left(x\right)$. The optimization problem under such set up would be
  (42) $$\begin{array}{ccc} minimize\;L(x) & = & -\dot{V}\left(x\right)\\ subject\;to\;V(x) & = & {\bar{V}}_{i}, \end{array}$$
  where ${\bar{V}}_{i}\in R$. This optimization problem would search along each contour $V\left(x\right)={\bar{V}}_{i}$ to find the maximum value of $\dot{V}$ in this contour.
2. Base on step 1, set L(x)==0 to find the ${\bar{V}}_{0}$, which means in this contour $V\left(x\right)={\bar{V}}_{0}$ the maximum value of $\dot{V}$ is 0.
3. Repeat steps 1 and 2 in the compact sets of g2 and g3; get a set of ${\bar{V}}_{0}\left(g_{2},g_{3}\right)$.
4. Define the boundary function of states, $g\left(x\right)=B_{x}\left({\bar{V}}_{0}\right)$, where ∥x∥=g(x).
5. Find the $min(B_{x}\left(V_{0}\right))$ in the set of ${\bar{V}}_{0}\left(g_{2},g_{3}\right)$, which give the states’ minimum bounds. Then, the optimal linearization coefficients, g2 and g3, are picked up.

### 5.2. Pitch-Yaw Angle Channel Stability Analysis

Let:

(43) $$\begin{array}{ccc} {\tilde{w}}_{s} & \overset{\Delta }{\;=} & w_{s}-w_{d} \end{array}$$

(44) $$\begin{array}{ccc} {\tilde{\Omega }}_{s} & \overset{\Delta }{\;=} & \Omega_{s}-\Omega_{d} \end{array}$$

(45) $$\begin{array}{ccc} {\tilde{\sigma }}_{s} & \overset{\Delta }{\;=} & {\hat{\sigma }}_{s}-\sigma_{s} \end{array}$$

(46) $$\begin{array}{ccc} \gamma_{0}\left(T_{s},\rho_{w}\right) & \overset{\Delta }{\;=} & \sqrt{n}∥\left(I-exp\left(A_{m}T_{s}\right)\right){A_{m}}^{-1}K_{s}∥\left(L\rho_{w}+L_{0}\right) \end{array}$$

(47) $$\begin{array}{ccc} \gamma_{1}\left(T_{s},\rho_{w},\rho_{\eta }\right) & \overset{\Delta }{\;=} & \sqrt{\lambda_{\max}\left[{\left(J_{s}A_{m}\right)}^{⊤}\left(J_{s}A_{m}\right)\right]}\gamma_{0}\left(T_{s},\rho_{w}\right)+2b_{d\sigma }T_{s}\sqrt{n} \end{array}$$

(48) $$\begin{array}{ccc} \gamma_{2}\left(T_{s},\rho_{w},\rho_{\eta }\right) & \overset{\Delta }{\;=} & \sqrt{n}\left|\right|{\left(sI-A_{m}\right)}^{-1}K_{s}|{|}_{L_{1}}\gamma_{1}\left(T_{s},\rho_{w},\rho_{\eta }\right) \end{array}$$

(49) $$\begin{array}{ccc} b_{d\sigma }\left(\rho_{w}\right) & \overset{\Delta }{\;=} & L_{1}\left({∥H_{s}\left(w\right)∥}_{L_{1}}+{∥K_{s}∥}_{L_{1}}\left(\rho_{\eta }+L\rho_{w}+L_{0}\right)\right)\\ & & +L_{2}\rho_{w}+L_{3} \end{array}$$

(50) $$\begin{array}{ccc} \rho_{f} & \overset{\Delta }{\;=} & {∥\left(C\left(s\right)-1\right)J_{s}∥}_{L_{1}}∥w_{s_{d}}∥+{∥1-C(s)∥}_{L_{1}}\left(L\rho_{w}+L_{0}\right)\\ & & +{∥C(s)∥}_{L_{1}}\gamma_{1}\left(T_{s},\rho_{w},\rho_{\eta }\right) \end{array}$$

**Lemma** **2.** *Considering the system described in (9) with the state predictor (12), adaptive law (15) and control law (16), if the truncated norm ${∥w_{s_{t_{1}}}∥}_{L_{\infty }}\leq \rho_{w}$, ${∥\eta_{t_{1}}∥}_{L_{\infty }}\leq \rho_{\eta }$, ${∥x_{t_{1}}∥}_{L\infty }\leq b_{x}$ for any time $t_{1}\geq 0$, we have*

(51) $$\begin{array}{c} {∥{\tilde{\sigma }}_{s_{t_{1}}}∥}_{\infty }\leq \gamma_{1}\left(T_{s},\rho_{w},\rho_{\eta }\right),{∥e_{t_{1}}∥}_{\infty }\leq \gamma_{2}\left(T_{s},\rho_{w},\rho_{\eta }\right)\;.\; \end{array}$$

**Proof of Lemma** **2.** The solution of system (13) in the time interval $\left[\left(i-1\right)T_{s},t+\left(i-1\right)T_{s}\right],t\in \left[0,T_{s}\right]$ is

(52) $$\begin{array}{ccc} e(\left(i-1\right)T_{s}+t) & = & exp\left(A_{m}t\right)e\left(\left(i-1\right)T_{s}\right)\\ & & +\int_{\left(i-1\right)T_{s}}^{\left(i-1\right)T_{s}+t}exp\left(A_{m}\left(\left(i-1\right)T_{s}+t-\tau \right)\right)K_{s}{\hat{\sigma }}_{s}\left(\left(i-1\right)T_{s}\right)d\tau \\ & & -\int_{\left(i-1\right)T_{s}}^{\left(i-1\right)T_{s}+t}exp\left(A(\left(i-1\right)T_{s}+t-\tau )\right)K_{s}\sigma_{s}\left(\tau \right)d\tau \;. \end{array}$$

When $t=T_{s}$, it follows from Equation (52) that

(53) $$\begin{array}{ccc} e(iT_{s}) & = & exp\left(A_{m}T_{s}\right)e\left(\left(i-1\right)T_{s}\right)\\ & & +\int_{\left(i-1\right)T_{s}}^{iT_{s}}exp\left(A_{m}\left(\left(i-1\right)T_{s}+T_{s}-\tau \right)\right)K_{s}{\hat{\sigma }}_{s}\left(\left(i-1\right)T_{s}\right)d\tau \\ & & -\int_{\left(i-1\right)T_{s}}^{iT_{s}}exp\left(A_{m}\left(\left(i-1\right)T_{s}+T_{s}-\tau \right)\right)K_{s}\sigma_{s}\left(\tau \right)d\tau . \end{array}$$

According to the choice of adaptive law in (15), we have

(54) $$\begin{array}{c} exp\left(A_{m}T_{s}\right)e\left(\left(i-1\right)T_{s}\right)+\int_{\left(i-1\right)T_{s}}^{iT_{s}}exp\left(A_{m}\left(iT_{s}-\tau \right)\right)K_{s}{\hat{\sigma }}_{s}\left(\left(i-1\right)T_{s}\right)d\tau =0\;. \end{array}$$

It follows from Equation (53) that

(55) $$\begin{array}{c} e\left(iT_{s}\right)=-\int_{\left(i-1\right)T_{s}}^{iT_{s}}exp\left(A(iT_{s}-\tau )\right)K_{s}\sigma_{s}\left(\tau \right)d\tau \;.\; \end{array}$$

The norm of it is

(56) $$\begin{array}{ccc} ∥e(iT_{S})∥ & = & ∥\int_{\left(i-1\right)T_{s}}^{iT_{s}}exp(A_{m}\left(iT_{s}-\tau \right))K_{s}\sigma_{s}\left(\tau \right)d\tau ∥\\ & \leq & \int_{\left(i-1\right)T_{s}}^{iT_{s}}∥exp(A_{m}\left(iT_{s}-\tau \right))K_{s}∥∥\sigma_{s}\left(\tau \right)∥d\tau \\ & \leq & \int_{\left(i-1\right)T_{s}}^{iT_{s}}∥exp(A_{m}\left(iT_{s}-\tau \right))K_{s}∥d\tau \sqrt{n}{∥\sigma_{s}\left(\tau \right)∥}_{\infty }\\ & = & ∥exp\left(A_{m}iT_{s}\right)exp(-A_{m}\tau ){|}_{\left(i-1\right)T_{s}}^{iT_{s}}A_{m}^{-1}K_{s}∥\sqrt{n}{∥\sigma_{s}\left(iT_{s}\right)∥}_{L_{\infty }}\\ & = & ∥\left(I-exp\left(A_{m}T_{s}\right)\right)A_{m}^{-1}K_{s}∥\sqrt{n}{∥\sigma_{s}\left(iT_{s}\right)∥}_{L_{\infty }}\;.\; \end{array}$$

Since $\sigma_{s}=I_{s}\sigma$, $\sigma_{s}$ is a subset of σ. Using Assumption 1 in (56), we could have

(57) $$\begin{array}{c} ∥e\left(iT_{s}\right)∥\leq ∥\left(I-exp\left(A_{m}T_{s}\right)\right)A_{m}^{-1}K_{s}∥\left(L{∥w_{s}\left(iT_{s}\right)∥}_{L_{\infty }}+L_{0}\right)\sqrt{n}\;. \end{array}$$

In the condition of this Lemma $\left|\right|w_{s_{t_{1}}}{\left|\right|}_{L_{\infty }}\leq \rho_{w}$, for all *i* while $iT_{s}<t_{1}$, Equation (57) could be

(58) $$\begin{array}{cc} & ∥e\left(iT_{s}\right){∥}_{\infty }\leq ∥\left(I-exp\left(A_{m}T_{s}\right)\right)A_{m}^{-1}K_{s}∥\left(L\rho_{x}+L_{0}\right)\sqrt{n}\;.\; \end{array}$$

Using the definition of $\gamma_{0}\left(T_{s},\rho_{x}\right)$ in (46), finally we could get

(59) $$\begin{array}{c} ∥e\left(iT_{s}\right){∥}_{\infty }\leq \gamma_{0}\left(T_{s},\rho_{x}\right)\;.\; \end{array}$$

For all $iT_{s}<t_{1}$, according to Assumption 2, the derivative of uncertainties could be written in

$$\begin{array}{ccc} ∥{\dot{\sigma }}_{s_{t_{1}}}{∥}_{L_{\infty }} & \leq & L_{1}∥{\dot{w}}_{s_{t_{1}}}{∥}_{L_{\infty }}+L_{2}{∥w_{s_{t_{1}}}∥}_{L_{\infty }}+L_{3}\\ & \leq & L_{1}∥{\left(H_{s}\left(w\right)+K_{s}\left(\eta_{s}+\sigma_{s}\right)\right)}_{t_{1}}{∥}_{L_{\infty }}+L_{2}{∥w_{s_{t_{1}}}∥}_{L_{\infty }}+L_{3}\\ & \leq & L_{1}(∥H_{s}{\left(w\right)∥}_{L_{1}}+∥K_{s}{∥}_{L_{1}}\left(\rho_{\eta }+L\rho_{w}+L_{0}\right))+L_{2}\rho_{w}+L_{3}\;.\; \end{array}$$

Using the definition of $b_{d\sigma }$ in (49), then

(60) $$\begin{array}{c} ∥{\dot{\sigma }}_{s_{t_{1}}}{∥}_{L_{\infty }}\leq b_{d\sigma }\;. \end{array}$$

It follows from (54) that

(61) $$\begin{array}{ccc} e(iT_{s}) & = & \left(I-exp\left(A_{m}T_{s}\right)\right)e\left(iT_{s}\right)-\int_{iT_{s}}^{\left(i+1\right)T_{s}}exp\left(A_{m}\left(\left(i+1\right)T_{s}-\tau \right)\right)K_{s}{\hat{\sigma }}_{s}\left(iT_{s}\right)d\tau \\ & = & -\int_{iT_{s}}^{\left(i+1\right)T_{s}}exp\left(A_{m}\left(\left(i+1\right)T_{s}-\tau \right)\right)A_{m}K_{s}e\left(iT_{s}\right)d\tau \\ & & -\int_{iT_{s}}^{\left(i+1\right)T_{s}}exp\left(A\left(\left(i+1\right)T_{s}-\tau \right)\right)K_{s}{\hat{\sigma }}_{s}\left(iT_{s}\right)d\tau \\ & = & -\int_{iT_{s}}^{\left(i+1\right)T_{s}}exp\left(A_{m}\left(\left(i+1\right)T_{s}-\tau \right)\right)K_{s}\left({\hat{\sigma }}_{s}\left(iT_{s}\right)+A_{m}e\left(iT\right)\right)d\tau \;. \end{array}$$

Hence, (55) and (61) imply that

(62) $$\begin{array}{ccc} \int_{\left(i-1\right)T_{s}}^{iT_{s}}exp\left(A_{m}\left(iT_{s}-\tau \right)\right)K_{s}\sigma_{s}\left(\tau \right)d\tau & = & \int_{iT_{s}}^{\left(i+1\right)T_{s}}exp\left(A_{m}\left(\left(i+1\right)T_{s}-\tau \right)\right)K_{s}({\hat{\sigma }}_{s}\left(iT_{s}\right)\\ & & +A_{m}e\left(iT_{s}\right))d\tau \;,\; \end{array}$$

and there exists $t_{p}\in \left[\left(i-1\right)T_{s},iT_{s}\right]$ such that

(63) $$K_{s}{\hat{\sigma }}_{s}\left(iT_{s}\right)+A_{m}e\left(iT_{s}\right)=K_{s}\sigma_{s}\left(t_{p}\right)\;.$$

For any $t<t_{1}$, there exists $t_{p}\in \left[\left(i-1\right)T_{s},iT_{s}\right]$ such that $|t-t_{p}|\leq 2T_{s}$ which satisfies (63),

(64) $$\begin{array}{ccc} ∥{\hat{\sigma }}_{s}\left(t\right)-\sigma_{s}\left(t\right)∥ & \leq & ∥{\hat{\sigma }}_{s}\left(t\right)-\sigma_{s}\left(t_{p}\right)∥+∥\sigma_{s}\left(t\right)-\sigma_{s}\left(t_{p}\right)∥\\ & \leq & ∥{\hat{\sigma }}_{s}\left(iT_{s}\right)-\sigma_{s}\left(t_{p}\right)∥+∥\sigma_{s}\left(t\right)-\sigma_{s}\left(t_{p}\right)∥\\ & \leq & J_{s}A_{m}∥e\left(iT_{s}\right)∥+\int_{t_{p}}^{t}∥B{\dot{\sigma }}_{s}\left(\tau \right)∥d\tau \;. \end{array}$$

The bound of ${\dot{\sigma }}_{s}\left(t\right)$ is derived in Equation (60). Then we have

(65) $$∥{\hat{\sigma }}_{s}\left(t\right)-\sigma_{s}\left(t\right)∥\leq \sqrt{\lambda_{\max}\left({\left(J_{s}A_{m}\right)}^{⊤}\left(J_{s}A_{m}\right)\right)}\gamma_{0}\left(T_{s},\rho_{x}\right)+2b_{d\sigma }T_{s}\sqrt{n}\;.\;$$

It follows from the definition of ${\tilde{\sigma }}_{s}\left(t\right)$ and $\gamma_{1}\left(T_{s},\rho_{w},\rho_{\eta }\right)$ in (45), (47) that

(66) $$\begin{array}{c} \left|\right|{\tilde{\sigma }}_{s_{t_{1}}}\left|\right|\leq \gamma_{1}\left(T_{s},\rho_{x},\rho_{\eta }\right)\;.\; \end{array}$$

Using the dynamics in Equation (13), we have

(67) $$\begin{array}{c} e\left(s\right)={\left(sI-A_{m}\right)}^{-1}K_{s}\left({\hat{\sigma }}_{s}\left(s\right)-\sigma_{s}\left(s\right)\right)\;. \end{array}$$

Hence,

(68) $$\begin{array}{ccc} \left|\right|e_{t_{1}}{\left|\right|}_{L_{\infty }} & \leq & \left|\right|{\left(sI-A_{m}\right)}^{-1}K_{s}{\left|\right|}_{L_{1}}\left|\right|{\left({\hat{\sigma }}_{s}-\sigma_{s}\right)}_{t}{\left|\right|}_{L_{\infty }},\\ & \leq & \left|\right|{\left(sI-A_{m}\right)}^{-1}K_{s}{\left|\right|}_{L_{1}}\gamma_{1}\left(T_{s},\rho_{w},\rho_{\eta }\right)\;.\; \end{array}$$

Using the norm property $\left|\right|e_{t_{1}}\left|\right|\leq \sqrt{n}\left|\right|e_{t_{1}}|{|}_{L_{\infty }}$, we have

(69) $$\begin{array}{c} \left|\right|e_{t_{1}}\left|\right|\leq \sqrt{n}\left|\right|{\left(sI-A_{m}\right)}^{-1}K_{s}{\left|\right|}_{L_{1}}\gamma_{1}\left(T_{s},\rho_{w},\rho_{\eta }\right) \end{array}$$

where *n* is the dimension of $w_{s}\left(t\right)$. The value of *n* is equal to 2 in this situation. It follows from the definition of $\gamma_{2}\left(T_{s},\rho_{w},\rho_{\eta }\right)$ in (48),

(70) $$\begin{array}{c} \left|\right|e_{t_{1}}\left|\right|\leq \gamma_{2}\left(T_{s},\rho_{w},\rho_{\eta }\right) \end{array}$$

which completes the proof. □

**Lemma** **3.** *For any given bounded $\rho_{w}$, $\rho_{\eta }$*

(71) $$\begin{array}{c} \lim_{T\to 0}\gamma_{0}\left(T_{s},\rho_{w}\right)\to 0 \end{array}$$

(72) $$\begin{array}{c} \lim_{T\to 0}\gamma_{1}\left(T_{s},\rho_{w},\rho_{\eta }\right)\to 0 \end{array}$$

(73) $$\begin{array}{c} \lim_{T\to 0}\gamma_{2}\left(T_{s},\rho_{w},\rho_{\eta }\right)\to 0\;.\; \end{array}$$

**Proof of Lemma** **3.** Recall the definition of $\gamma_{0}\left(T_{s},\rho_{w}\right)$ in (46), the insides of the integration are functions with bounded values. As $T_{s}\to 0$ we can get $\gamma_{0}\left(T_{s},\rho_{w}\right)\to 0$.

Similarly the limits of $\gamma_{1}\left(T_{s},\rho_{w},\rho_{\eta }\right)$ and $\gamma_{2}\left(T_{s},\rho_{w},\rho_{\eta }\right)$ going to zero could be proved. The proof is completed. □

**Lemma** **4.** *Considering the system described in (9) with the state predictor (12), adaptive law (15) and control law (16), if the truncated norm ${∥w_{s_{t_{1}}}∥}_{L_{\infty }}\leq \rho_{w}$, ${∥\eta_{s_{t_{1}}}∥}_{L_{\infty }}\leq \rho_{\eta }$, ${∥x_{t_{1}}∥}_{L\infty }\leq b_{x}$, $∥\Omega_{s_{t_{1}}}∥\leq \rho_{\Omega }$ for any time $t_{1}\geq 0$, we have*

(74) $$\begin{array}{c} ∥{\tilde{\Omega }}_{s_{t_{1}}}∥\leq \sqrt{C},\;∥{\tilde{w}}_{s_{t_{1}}}∥\leq \sqrt{C} \end{array}$$

*where $C\overset{\Delta }{\;=}\max\left\{\frac{\rho_{f}^{2}}{\lambda_{\min}\left(A_{m2}^{T}A_{m2}\right)},\frac{\rho_{f}^{2}}{\sqrt{\lambda_{\min}\left(A_{m1}^{T}A_{m1}\right)}\sqrt{\lambda_{\min}\left(A_{m2}^{T}A_{m2}\right)}}\right\}$*.

**Proof of Lemma** **4.** Considering this Lyapunov function candidate,

(75) $$\begin{array}{c} V=\frac{1}{2}\left({\tilde{\Omega }}_{s}^{T}{\tilde{\Omega }}_{s}+{\tilde{w}}_{s}^{T}{\tilde{w}}_{s}\right)\;,\; \end{array}$$

first, we prove that

(76) $$\begin{array}{c} V(t)\leq C\;.\; \end{array}$$

Based on the control laws in (16) and the definition in (20) (43) and (44), the derivative of (75) is:

(77) $$\begin{array}{ccc} \dot{V} & = & {\dot{\tilde{\Omega }}}_{s}^{T}{\tilde{\Omega }}_{s}+{\dot{\tilde{w}}}_{s}^{T}{\tilde{w}}_{s}\\ & = & {\left(\Psi_{s}w_{s}-{\dot{\Omega }}_{d}\right)}^{T}{\tilde{\Omega }}_{s}+{\left(H_{s}\left(w\right)+K_{s}\left(\eta +\sigma_{s}\right)-{\dot{w}}_{d}\right)}^{T}{\tilde{w}}_{s}\\ & = & {\left(\Psi_{s}w_{d}-{\dot{\Omega }}_{d}\right)}^{T}{\tilde{\Omega }}_{s}+{\left(\Psi_{s}{\tilde{w}}_{s}\right)}^{T}{\tilde{\Omega }}_{s}+{\left(H_{s}\left(w\right)+K_{s}\left(\eta_{s}+\sigma_{s}\right)-{\dot{w}}_{d}\right)}^{T}\tilde{w}\\ & = & {{\tilde{\Omega }}_{s}}^{T}A_{m1}{\tilde{\Omega }}_{s}+{{\tilde{\Omega }}_{s}}^{T}\left(\Psi_{s}{\tilde{w}}_{s}\right)+{\left(H_{s}\left(w\right)+K_{s}\left(\eta_{s}+\sigma_{s}\right)-{\dot{w}}_{d}\right)}^{T}{\tilde{w}}_{s}\\ & = & {{\tilde{\Omega }}_{s}}^{T}A_{m1}{\tilde{\Omega }}_{s}+{\left(\Psi_{s}^{T}{\tilde{\Omega }}_{s}+H_{s}\left(w\right)+K_{s}\left(\eta_{s}+\sigma_{s}\right)-{\dot{w}}_{d}\right)}^{T}{\tilde{w}}_{s}\\ & = & {{\tilde{\Omega }}_{s}}^{T}A_{m1}{\tilde{\Omega }}_{s}+{{\tilde{w}}_{s}}^{T}A_{m2}{\tilde{w}}_{s}+{\left[\left(C\left(s\right)-1\right)J_{s}{\dot{w}}_{d}+\left(1-C\left(s\right)\right)\sigma_{s}-C\left(s\right){\tilde{\sigma }}_{s}\right]}^{T}{\tilde{w}}_{s}\\ & = & {{\tilde{\Omega }}_{s}}^{T}A_{m1}{\tilde{\Omega }}_{s}+{\tilde{w}}_{s}^{T}A_{m2}{\tilde{w}}_{s}+f\left(t,w_{s}\right){\tilde{w}}_{s} \end{array}$$

where $A_{m1}$ and $A_{m2}$ are Hurwitz matrices, which ensure that the total of the first two quadratic terms would be negative.

For $f\left(t,w_{s}\left(t\right)\right)={\left[\left(C\left(s\right)-1\right)J_{s}{\dot{w}}_{d}+\left(1-C\left(s\right)\right)\sigma_{s}-C\left(s\right){\tilde{\sigma }}_{s}\right]}^{T}$, according to Assumption 1 and Lemma 1,

(78) $$\begin{array}{ccc} {∥f(t,w_{s}\left(t\right))∥}_{\infty } & \leq & ∥\left(C\left(s\right)-1\right)J_{s}{s∥}_{L_{1}}∥w_{d}{∥+∥1-C\left(s\right)∥∥\sigma \left(t\right)}_{s}∥+∥C\left(s\right)∥∥{\tilde{\sigma }}_{s}∥\\ & \leq & ∥\left(C\left(s\right)-1\right)J_{s}{s∥}_{L_{1}}∥w_{d}{∥+∥1-C\left(s\right)∥}_{L_{1}}\left(L\rho_{w}+L_{0}\right)\\ & & +{∥C\left(s\right)∥}_{L_{1}}\gamma_{1}\left(T_{s},\rho_{w},\rho_{\eta }\right) \end{array}$$

By the definition in (50), thus

(79) $$\begin{array}{c} ∥f\left(t,w_{s}\left(t\right)\right)∥\leq \rho_{f}\;.\; \end{array}$$

Next, we prove Equation (76) by contradiction method. Assuming the opposite of Equation (76) is true. Then if at any time $t_{1}>0$, one has $V(t_{1})>C$. According to (75), we could have $∥{\tilde{\Omega }}_{s}∥>\sqrt{C}$ or $∥{\tilde{w}}_{s}∥>\sqrt{C}$.

Based on this, two-case discussions under this are presented as follows,

**Case 1.** For $∥{\tilde{w}}_{s}∥>\sqrt{C}$,thus in (77), we could get

(80) $$\begin{array}{ccc} \dot{V} & = & {\tilde{\Omega }}_{s}^{T}A_{m1}{\tilde{\Omega }}_{s}+{\tilde{w}}_{s}^{T}A_{m2}{\tilde{w}}_{s}+K_{s}f\left(t,w_{s}\right){\tilde{w}}_{s}\\ & \leq & {\tilde{\Omega }}_{s}^{T}A_{m1}{\tilde{\Omega }}_{s}+{\tilde{w}}_{s}^{T}A_{m2}{\tilde{w}}_{s}+\rho_{f}{\tilde{w}}_{s}\\ & \leq & {\tilde{\Omega }}_{s}^{T}A_{m1}{\tilde{\Omega }}_{s}-{∥\lambda_{\min}\left(A_{m2}\right)∥}_{L_{2}}{∥{\tilde{w}}_{s}∥}_{L_{2}}^{2}+\rho_{f}{\tilde{w}}_{s} \end{array}$$

which could easily find $\dot{V}\left(t_{1}\right)<0$.

**Case 2.** For $∥{\tilde{\Omega }}_{s}∥>\sqrt{C}$

If ${\tilde{w}}_{s}\leq \sqrt{C}$, from (77),

(81) $$\begin{array}{ccc} \dot{V} & = & {\tilde{\Omega }}_{s}^{T}A_{m1}{\tilde{\Omega }}_{s}+{\tilde{w}}_{s}^{T}A_{m2}{\tilde{w}}_{s}+K_{s}f\left(t,w_{s}\right)\tilde{w}\\ & \leq & {\tilde{\Omega }}_{s}^{T}A_{m1}{\tilde{\Omega }}_{s}+{\tilde{w}}_{s}^{T}A_{m2}{\tilde{w}}_{s}+\rho_{f}{\tilde{w}}_{s}\\ & \leq & \left(-{∥\lambda_{\min}\left(A_{m1}\right)∥}_{L_{2}}{∥{\tilde{\Omega }}_{s}∥}_{L_{2}}^{2}+\rho_{f}{\tilde{w}}_{s}\right)+{\tilde{w}}_{s}^{T}A_{m2}{\tilde{w}}_{s}, \end{array}$$

based on the conditions settings for this case, we could get $\dot{V}\left(t\right)<0$.

If ${\tilde{w}}_{s}>\sqrt{C}$, according to Case 1, we could easily verify $\dot{V}<0$.

Hence, if $V(t_{1})>C$, then from the above Cases 1 and 2 we have

(82) $$\begin{array}{c} \dot{V}\left(t_{1}\right)<0\;.\; \end{array}$$

For t=0, it means that if V(0)>C, V(t) will keep decreasing until V(t)≤C. If V(0)≤C, then V(t)≤C.

Thus, for all $t_{1}\geq 0$, V(t)<C. Since ${∥{\tilde{w}}_{s_{t_{1}}}∥}^{2}={\tilde{w}}_{s}^{T}{\tilde{w}}_{s}\leq V\left(t\right),{∥{\tilde{\Omega }}_{s_{t_{1}}}∥}^{2}={\tilde{\Omega }}_{s}^{T}{\tilde{\Omega }}_{s}\leq V\left(t\right)$, we have

(83) $$\begin{array}{c} ∥{\tilde{\Omega }}_{s_{t_{1}}}∥\leq \sqrt{C},\;\;∥{\tilde{w}}_{s_{t_{1}}}∥\leq \sqrt{C},\;\;\forall \;t_{1}\geq 0\;.\; \end{array}$$

 □

**Lemma** **5.** *For the system in (2), (6) with the $L_{1}$ backstepping adaptive controller in (16), if the truncated $L_{\infty }$ norm ${∥\Omega_{s_{t_{1}}}∥}_{L_{\infty }}\leq \rho_{\Omega }$, ${∥w_{s_{t_{1}}}∥}_{L_{\infty }}\leq \rho_{w}$, ${∥x_{t_{1}}∥}_{L\infty }\leq b_{x}$ and ${∥\eta_{s_{t_{1}}}∥}_{L_{\infty }}\leq \rho_{\eta }$ for any time $t_{1}\geq 0$, then*

(84) $$\begin{array}{ccc} {∥\eta_{s_{t_{1}}}∥}_{L_{\infty }} & \leq & \sqrt{C}∥J_{s}A_{m2}∥+{∥J_{s}H_{s}\left(w\right)∥}_{L_{1}}+\sqrt{C}{∥J_{s}\Psi_{s}^{T}\left(\Omega \right)∥}_{L_{1}}\\ & & +{∥C(s)∥}_{L_{1}}\left(L\rho_{w}+L_{0}\right)+{∥C\left(s\right)J_{s}s∥}_{L_{1}}∥w_{d}∥\\ & & +{∥C(s)∥}_{L_{1}}\gamma_{1}\left(T_{s},\rho_{w},\rho_{\eta }\right) \end{array}$$

*and*

(85) $$\begin{array}{ccc} {∥w_{s_{t_{1}}}∥}_{L_{\infty }} & \leq & ∥w_{d}∥+\sqrt{C} \end{array}$$

(86) $$\begin{array}{ccc} {∥\Omega_{s_{t_{1}}}∥}_{L_{\infty }} & \leq & ∥\Omega_{d}∥+\sqrt{C}\;.\; \end{array}$$

**Proof of Lemma** **5.** According to (16),

(87) $$\begin{array}{ccc} \eta_{s}\left(s\right) & = & \eta_{b}\left(s\right)+\eta_{a}\left(s\right)\\ & = & J_{s}\left(A_{m2}\tilde{w}-H_{s}\left(w\right)-\Psi_{s}^{T}\left(\Omega \right){\tilde{\Omega }}_{s}\right)+C\left(s\right)\left(\sigma_{s}-J_{s}sw_{d}+{\tilde{\sigma }}_{s}\right), \end{array}$$

taking the norm of this equation, we could have

(88) $$\begin{array}{ccc} {∥\eta_{s_{t_{1}}}∥}_{L_{\infty }} & \leq & ∥J_{s}A_{m2}∥{∥{\tilde{w}}_{s}∥}_{L_{\infty }}+{∥J_{s}H_{s}\left(w\right)∥}_{L_{1}}+{∥J_{s}\Psi_{s}^{T}\left(\Omega \right)∥}_{L_{1}}{∥{\tilde{\Omega }}_{s}∥}_{L_{\infty }}\\ & & +{∥C(s)∥}_{L_{1}}∥\sigma_{s}∥+{∥C\left(s\right)J_{s}s∥}_{L_{1}}∥w_{d}∥+{∥C(s)∥}_{L_{1}}{∥{\tilde{\sigma }}_{s}∥}_{\infty }\;.\; \end{array}$$

Use Assumption 1, Lemmas 1 and 4, we could get

(89) $$\begin{array}{ccc} {∥\eta_{t_{1}}∥}_{L_{\infty }} & \leq & \sqrt{C}∥J_{s}A_{m2}∥+{∥J_{s}H_{s}\left(w\right)∥}_{L_{1}}+\sqrt{C}{∥J_{s}\Psi_{s}^{T}\left(\Omega \right)∥}_{L_{1}}\\ & & +{∥C(s)∥}_{L_{1}}\left(L\rho_{w}+L_{0}\right)+{∥C\left(s\right)J_{s}s∥}_{L_{1}}∥w_{d}∥\\ & & +{∥C(s)∥}_{L_{1}}\gamma_{1}\left(T_{s},\rho_{w},\rho_{\eta }\right)\;.\; \end{array}$$

Based on the definition of ${\tilde{w}}_{s}$ in (43)

(90) $$\begin{array}{c} w_{s}=w_{d}+{\tilde{w}}_{s}\;.\; \end{array}$$

Thus, we could get

(91) $$\begin{array}{ccc} {∥w_{s}∥}_{L_{\infty }} & \leq & ∥w_{d}∥+{∥{\tilde{w}}_{s}∥}_{L_{\infty }}\\ & \leq & ∥w_{d}∥+\sqrt{C}\;.\; \end{array}$$

Same for $\Omega_{s}$,

(92) $$\begin{array}{c} {∥\Omega_{s}∥}_{L_{\infty }}\leq ∥\Omega_{d}∥+\sqrt{C}\;.\; \end{array}$$

 □

**Lemma** **6.** *There exist $\rho_{w}>0$, $\rho_{\Omega }>0$, $\rho_{\eta }>0$ and $T_{s}>0$ such that*

(93) $$\begin{array}{c} \sqrt{C}∥J_{s}A_{m2}∥+{∥J_{s}H_{s}\left(w\right)∥}_{L_{1}}+\sqrt{C}{∥J_{s}\Psi_{s}^{T}\left(\Omega \right)∥}_{L_{1}}+{∥C(s)∥}_{L_{1}}\left(L\rho_{w}+L_{0}\right)\\ +{∥C\left(s\right)J_{s}s∥}_{L_{1}}∥w_{d}∥+{∥C(s)∥}_{L_{1}}\gamma_{1}\left(T_{s},\rho_{w},\rho_{\eta }\right)<\rho_{\eta } \end{array}$$

*and*

(94) $$\begin{array}{ccc} ∥w_{d}∥+\sqrt{C} & < & \rho_{w} \end{array}$$

(95) $$\begin{array}{ccc} ∥\Omega_{d}∥+\sqrt{C} & < & \rho_{\Omega }\;.\; \end{array}$$

**Proof of Lemma** **6.** Let us choose $\rho_{\eta }$ such that

(96) $$\begin{array}{ccc} \rho_{\eta } & = & \sqrt{C}∥J_{s}A_{m2}∥+{∥J_{s}H_{s}\left(w\right)∥}_{L_{1}}+\sqrt{C}{∥J_{s}\Psi_{s}^{T}\left(\Omega \right)∥}_{L_{1}}\\ & & +{∥C(s)∥}_{L_{1}}\left(L\rho_{w}+L_{0}\right)+{∥C\left(s\right)J_{s}s∥}_{L_{1}}∥w_{d}∥+\Delta_{1} \end{array}$$

where $\Delta_{1}>0$ is any positive constant. From Lemma 5.2, there exists some $T_{s}$ to make

(97) $$\begin{array}{c} {∥C(s)∥}_{L_{1}}\gamma_{1}\left(T_{s},\rho_{w},\rho_{\eta }\right)<\Delta_{1}\;. \end{array}$$

For (94) and (95), we could get the value of the left sides. There must exist a set of $\rho_{w}$ and $\rho_{\Omega }$, which could satisfy the inequalities. □

**Theorem** **1.** For the system in (2), (6) with the $L_{1}$ backstepping adaptive controller in (16), choosing $T_{S}$ to make Lemma 6 hold, if ${∥x_{t_{1}}∥}_{L\infty }\leq b_{x}$ then

(98) $$\begin{array}{c} \begin{array}{ccc} {∥w_{s}∥}_{L_{\infty }}<\rho_{w}, & {∥\Omega_{s}∥}_{L_{\infty }}<\rho_{\Omega }, & {∥\eta_{s}∥}_{L_{\infty }}<\rho_{\eta }\;.\; \end{array} \end{array}$$

**Proof of Theorem** **1.** For t=0, it satisfy that

(99) $$\begin{array}{c} \begin{array}{ccc} w_{s}\left(0\right)<\rho_{w}, & \Omega_{s}\left(0\right)<\rho_{\Omega }, & \eta_{s}\left(0\right)<\rho_{\eta } \end{array}\;.\; \end{array}$$

Using proof by contradiction, assume Theorem 1 is not true, since $w_{s}\left(t\right)$, $\Omega_{s}\left(t\right)$ and $\eta_{s}\left(t\right)$ are continuous. There exists some $t^{\prime }\geq 0$ where

(100) $$\begin{array}{c} \begin{array}{ccc} {∥w_{s}∥}_{\infty }=\rho_{w}\;or & {∥\Omega_{s}∥}_{\infty }=\rho_{\Omega }\;or & {∥\eta_{s}∥}_{\infty }=\rho_{\eta }\;. \end{array} \end{array}$$

Thus,

(101) $$\begin{array}{c} \begin{array}{ccc} {∥w_{s}∥}_{L_{\infty }}\leq \rho_{w}, & {∥\Omega_{s}∥}_{L_{\infty }}\leq \rho_{\Omega }, & {∥\eta_{s}∥}_{L_{\infty }}\leq \rho_{\eta }\;. \end{array} \end{array}$$

Letting $t_{1}=t^{\prime }$, following from Lemmas 5 and 6 that

(102) $$\begin{array}{ccc} {∥w_{s_{t^{\prime }}}∥}_{L_{\infty }} & \leq & ∥w_{d}∥+\sqrt{C}\\ & < & \rho_{w} \end{array}$$

(103) $$\begin{array}{ccc} {∥\Omega_{s_{t^{\prime }}}∥}_{L_{\infty }} & \leq & ∥\Omega_{d}∥+\sqrt{C}\\ & < & \rho_{\Omega } \end{array}$$

and

(104) $$\begin{array}{ccc} {∥\eta_{s_{t^{\prime }}}∥}_{L_{\infty }} & \leq & \sqrt{C}∥J_{s}A_{m2}∥+{∥J_{s}H_{s}\left(w\right)∥}_{L_{1}}+\sqrt{C}{∥J_{s}\Psi_{s}^{T}\left(\Omega \right)∥}_{L_{1}}\\ & & +{∥C(s)∥}_{L_{1}}\left(L\rho_{w}+L_{0}\right)+{∥C\left(s\right)J_{s}s∥}_{L_{1}}∥w_{d}∥\\ & & +{∥C(s)∥}_{L_{1}}\gamma_{1}\left(T_{s},\rho_{w},\rho_{\eta }\right)\\ & < & \rho_{\eta }.\; \end{array}$$

These contradict what we get in (101) with the assumptions in (100). Therefore $t^{\prime }$ does not exist. Thus the statement in Theorem 1 holds for all t>0. □

### 5.3. Overall Stability Analysis

**Theorem** **2.** *If we choose $\Omega_{d}$ and design parameters $A_{m1}$, $A_{m2}$ to make $\rho_{w}$ and $\rho_{\Omega }$ satisfy the following equation,*

(105) $$\begin{array}{cc} i & \;\;∥{d_{1}}_{t_{1}}∥\leq b_{d1}\;∥{d_{2}}_{t_{1}}∥\leq b_{d2}\;. \end{array}$$

(106) $$\begin{array}{cc} ii & \;\;{\dot{V}}_{roll}\left(x\right)\leq 0,\;\forall x\in \left\{x|\;V_{roll}\left(x\right)=b_{x}^{2}\lambda_{\min}\left(P\right)\right\} \end{array}$$

(107) $$\begin{array}{cc} iii & \;\;∥x(0)∥\leq b_{x}\;,\;∥w_{s}\left(0\right)∥<\rho_{w}\;,\;∥\Omega_{s}\left(0\right)∥<\rho_{\Omega }\; \end{array}$$

*where $d_{1}$ and $d_{2}$ are defined in (24) and (25), then the entire system is stable and*

(108) $$\begin{array}{c} {∥x∥}_{L_{\infty }}\leq b_{x}\;,\;{∥w_{s}∥}_{L_{\infty }}<\rho_{w}\;,\;{∥\Omega_{s}∥}_{L_{\infty }}<\rho_{\Omega }\;. \end{array}$$

**Proof of Theorem** **2.** We prove this theorem by a contradiction argument, for t=0, it satisfies that

(109) $$\begin{array}{c} \begin{array}{ccc} ∥x(0)∥\leq b_{x}, & ∥w_{s}\left(0\right)∥<\rho_{w}, & ∥\Omega_{s}\left(0\right)∥<\rho_{\Omega } \end{array}\;.\; \end{array}$$

Assume Theorem 2 is not true, since x(t), $w_{s}\left(t\right)$ and $\Omega_{s}\left(t\right)$ are continuous. There exists some $t^{\prime }\geq 0$ where

(110) $$\begin{array}{c} \begin{array}{ccc} {∥w_{s}\left(t^{\prime }\right)∥}_{\infty }=\rho_{w}\;or & {∥\Omega_{s}\left(t^{\prime }\right)∥}_{\infty }=\rho_{\Omega } & {while∥x(t^{\prime })∥}_{\infty }\leq b_{x}\;. \end{array} \end{array}$$

Thus,

(111) $$\begin{array}{c} \begin{array}{ccc} {∥x_{t^{\prime }}∥}_{L_{\infty }}\leq b_{x}, & {∥w_{s_{t^{\prime }}}∥}_{L_{\infty }}\leq \rho_{w}, & {∥\Omega_{s_{t^{\prime }}}∥}_{L_{\infty }}\leq \rho_{\Omega }\;.\; \end{array} \end{array}$$

It follows from Theorem 1 that Equation (98) holds and contradicts with Equation (110). Therefore $t^{\prime }$ does not exist. Thus, the statement in Theorem 2 holds for all t>0. □

**Remark** **2.** *The conditions (105), (106) and (107) of Theorem 2 are feasible. In the definition of $d_{1}$ and $d_{2}$ we can readily obtain that $∥d_{1}∥\leq ∥s\theta t\theta +c\phi t\theta ∥\rho_{\omega }$ and $∥d_{1}∥\leq ∥J_{11}^{-1}\left(J_{33}-J_{22}\right)∥{\rho_{\omega }}^{2}+2L\rho_{\omega }+L_{0}$ noticing that $\sigma_{1}$ is subject to Assumption 1. By reducing the pitch angle operating range θ and the desired angular velocity $\rho_{\omega }$, we can always satisfy (107). However, this also limits the operation envelop of the vehicle. The control parameters and reference input should not violate the conditions. The simulation results in next section also validating the feasibility condition of Theorem 2*.

## 6. Simulation Results

### 6.1. Simulation for Roll Angle Stability Analysis

Following the optimum linearization procedure in Remark 1, the numerical solution for optimum linearization is $g_{2}=0.89$ and $g_{3}=1.85$. The parameter settings for the simulation are

$$\begin{array}{ccc} g_{1} & = & 2\\ Q & = & \left[\begin{array}{cc} 1 & 0.8\\ 0.5 & 1.8 \end{array}\right]\\ d_{1} & = & 0.1,\;d_{2}=0.1\;.\; \end{array}$$

**Figure 3:**
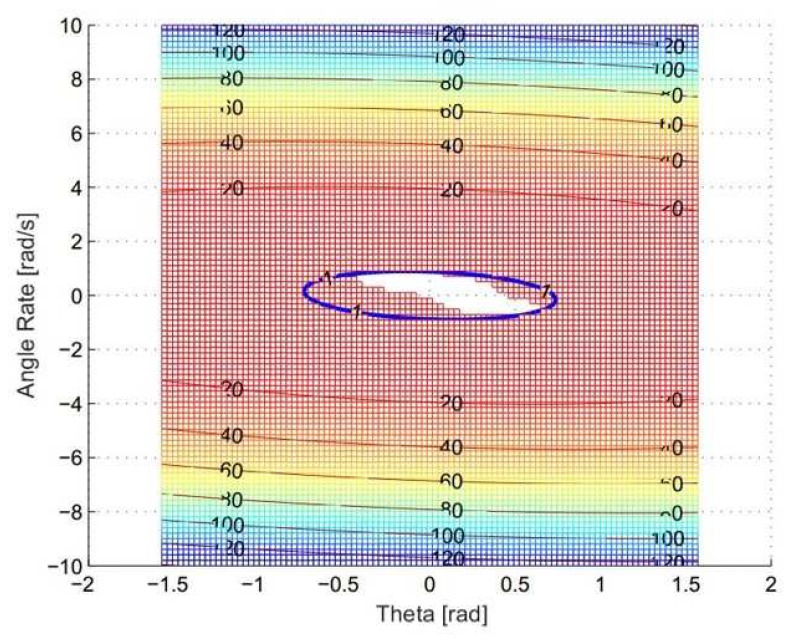
Roll channel stability.

The simulation results are shown in Figure 3. The region of $\dot{V}>0$ has been reduced by picking up the suitable coefficients g2 and g3. The blue contour is the one who has $max(\dot{V})=0$. The purple line give the bounds of ϕ and $w_{x}$, which are ϕ∈[−0.7392,0.7392] and $w_{x}\in \left[-0.8987,0.8987\right]$.

### 6.2. Pith-Yaw Angle Attitude Control

#### 6.2.1. Simulation Structure

The control law generated by the controller is a desired moment which can not be directly taken by the AUV. A dynamic control allocation module is brought into consideration to distribute the moment among these four thrusters. Similar to the study of quadcopters in [20], the total trust and moment provided by this thruster configuration with 4 thrusters can be expressed as the vector sum of the force and moment from each individual thruster. Using $w_{i}$ to represent each rotor’s speed, the trust provided by the $i_{th}$ thruster is

(112) $$\begin{array}{c} T_{i}=b\omega_{i}^{2}, \end{array}$$

where *b* is the truster coefficient. Thus, the total trust is given by

(113) $$\begin{array}{ccc} T_{b} & = & \sum_{i=1}^{4}T_{i}\\ & = & b(\omega_{1}^{2}+\omega_{2}^{2}+\omega_{3}^{2}+\omega_{4}^{2}). \end{array}$$

Pairwise differences in rotors’ speed drive the vehicle to rotate. The torques about the AUV’s y-axis and z-axis are generated by the moments

(114) $$\begin{array}{c} \tau_{y}=lb\left(-\omega_{1}^{2}-\omega_{2}^{2}+\omega_{3}^{2}+\omega_{4}^{2}\right) \end{array}$$

(115) $$\begin{array}{c} \tau_{z}=lb\left(\omega_{1}^{2}-\omega_{2}^{2}-\omega_{3}^{2}+\omega_{4}^{2}\right), \end{array}$$

where *l* is the distance from the thruster axis to the center of mass. Thus, the total trust and moments in the body frame are

(116) $$\begin{array}{c} \left[\begin{array}{c} T_{b}\\ \tau_{y}\\ \tau_{z} \end{array}\right]=\left[\begin{array}{cccc} -b & -b & -b & -b\\ -lb & -lb & lb & lb\\ lb & -lb & -lb & lb \end{array}\right]\left[\begin{array}{c} \omega_{1}^{2}\\ \omega_{2}^{2}\\ \omega_{3}^{2}\\ \omega_{4}^{2} \end{array}\right]=A\left[\begin{array}{c} \omega_{1}^{2}\\ \omega_{2}^{2}\\ \omega_{3}^{2}\\ \omega_{4}^{2} \end{array}\right]. \end{array}$$

**Figure 4:**
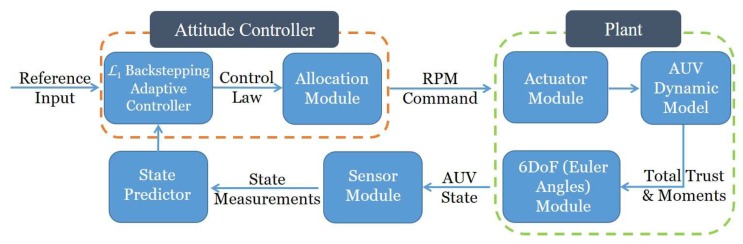
Simulation structure.

The pseudoinverse is used to calculate the allocation matrix $A^{-1}$. Then, by using

(117) $$\begin{array}{c} \left[\begin{array}{c} \omega_{1}^{2}\\ \omega_{2}^{2}\\ \omega_{3}^{2}\\ \omega_{4}^{2} \end{array}\right]=A^{-1}\left[\begin{array}{c} T_{b}\\ \tau_{y}\\ \tau_{z} \end{array}\right], \end{array}$$

the desired moments command could be distributed into RPM commands for each thruster. The simulation structure is shown in Figure 4.

#### 6.2.2. Closed-Loop Response

**Figure 5:**
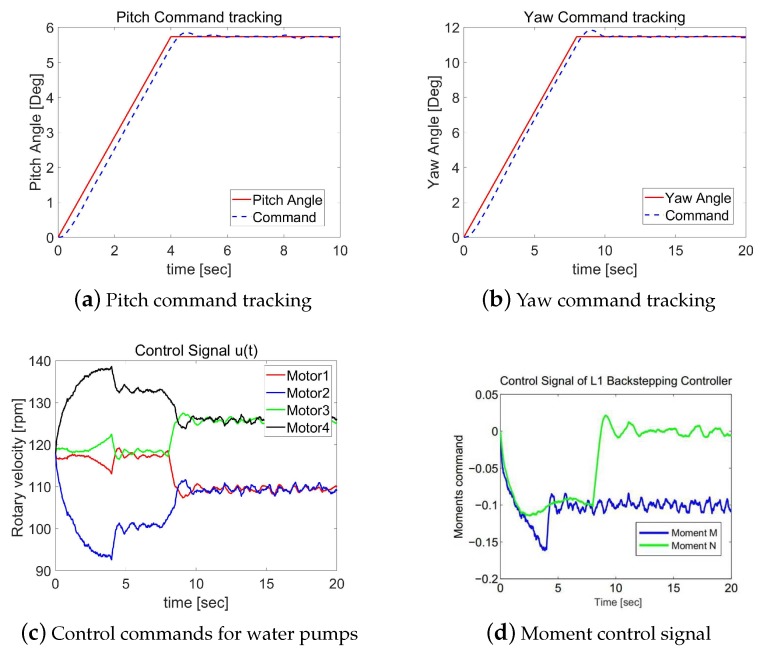
Case 1: Simulation results of the $L_{1}$ backstepping control.

**Figure 6:**
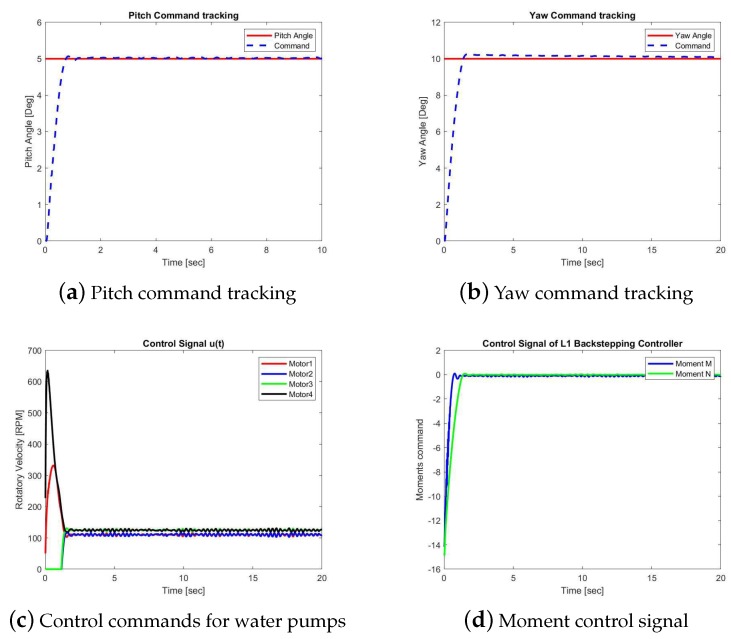
Case 2A: Simulation results of the $L_{1}$ backstepping control.

**Figure 7:**
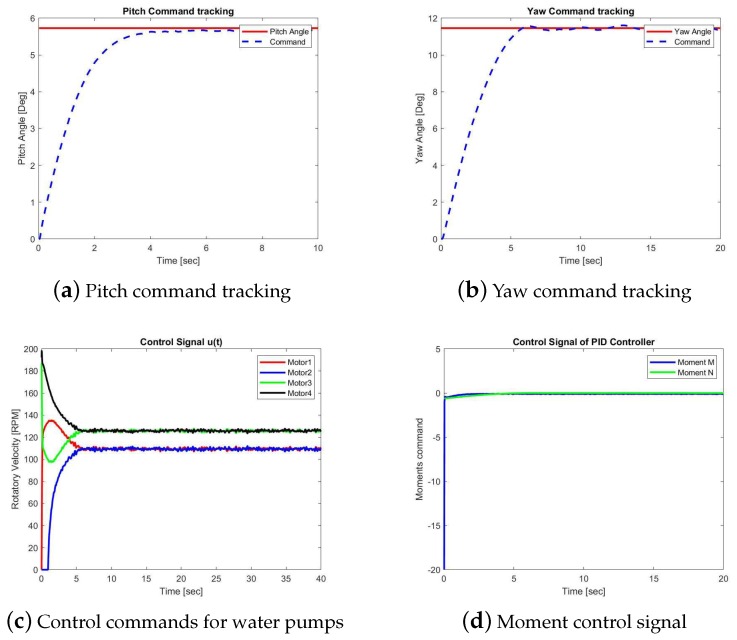
Case 2B: Simulation results of the PID control.

In this section, we will present the performance of two control methods. One is the proposed $L_{1}$ backstepping control, while the other is the PID control. Case 1 presents the simulation results with the designed reference inputs shown in Figure 5. Case 2 provides the simulation results with step functions as reference signals. The closed-loop responses of the $L_{1}$ backstepping control in Case 2 are shown in Figure 6a,b, while Figure 7a,b are for the PID method.

The control laws, $\eta_{y}$ and $\eta_{z}$, generated by these two controllers are shown in Figure 6c and Figure 7c, respectively, which are the desired moments with respect to axis y and axis z, namely, moment *M* and *N*. The RPM commands sent to the four water pumps are shown in Figure 6c and Figure 7c. Comparing Figure 6a,b, we could see the pitch angle uses less time than yaw angle to achieve the goal. When the desired angle of the pitch channel is reached, the commands are switched to put more effort into the yaw angle channel function.

## 7. Conclusions

In this paper, a robust $L_{1}$ backstepping attitude control has been proposed for AUVs in a dynamic environment. Moreover, a Lyapunov function-based optimum linearization method is presented to analyze the stability of the roll angle in the operation region without active stabilization. Simulation results have been provided to show the effectiveness of the proposed approach. Further research will focus on the performance improvement and precise trajectory-following algorithm design, which eventually will be extended to a fully autonomous underwater robotic network. 

## Figures and Tables

**Table 1:** Definitions and Notation.

Symbol	Definition	Symbol	Definition
ϕ	Roll angle	$\sigma_{s}$	Uncertainty in pitch and yaw channel
θ	Pitch angle	$\hat{\sigma }$	Estimation of $\sigma_{s}$
ψ	Yaw angle	$\eta_{b}$	Moment command from baseline controller
$w_{x}$	Roll rate	$\eta_{a}$	Moment command from adaptive controller
$w_{y}$	Pitch rate	$\Omega_{s}$	${\left[\begin{array}{cc} \theta & \psi \end{array}\right]}^{T}$
$w_{z}$	Yaw rate	$\Omega_{d}$	${\left[\begin{array}{cc} \theta_{d} & \psi_{d} \end{array}\right]}^{T}$
s*	sin(*)	$w_{s}$	${\left[\begin{array}{cc} x_{y} & w_{z} \end{array}\right]}^{T}$
c*	cos(*)	*J*	Moment of inertia of the AUV
t*	tan(*)	*K*	$J^{-1}$

**Figure A1:**
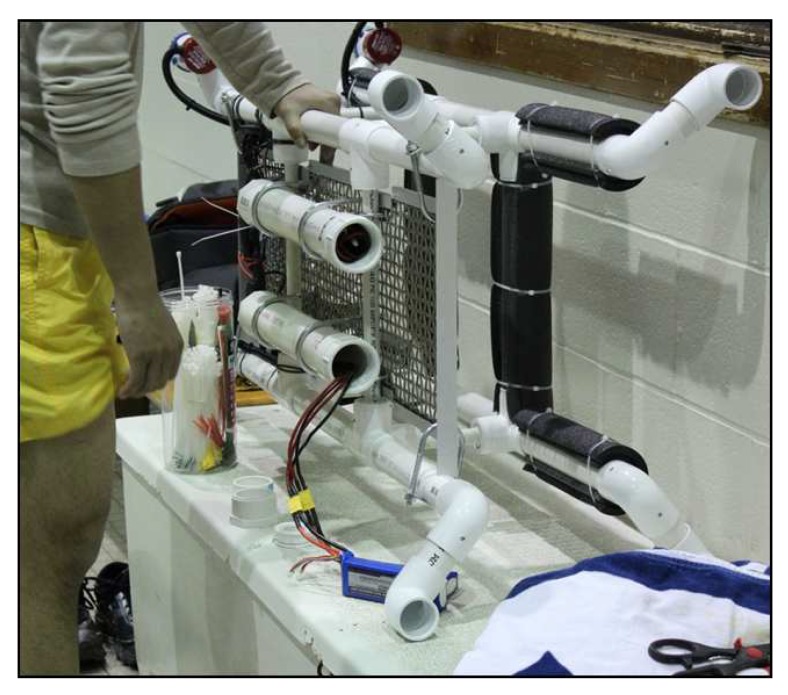
Prototype side view [9].

## Appendix A. AUV Modeling

The design of the prototype used in this paper is presented in Section 2. In this section, we would give more modeling details.

### Appendix A.1. Proof of Concept Testing

The proof of concept system is designed to be passively stable. In initial testing, this criterion has been sufficiently met with a self-righting time of approximately 1 second from a complete roll over. Initial testing of the propulsion system in a swimming pool environment has displayed an acceptable cruising speed and turn radius.

The fluid level and position of the buoyancy control unit is adjustable as shown in Figure A1. With all motors’ power set to zero, we adjust the fluid level to make the buoyancy force equal. Additionally, the position of the buoyancy is also adjusted to make the attitude of the AUV straight in the water.

This static balancing and alignment can make the vehicle naturally closed to the desired system. Although, the control law from the closed-loop controller can compensate these unbalanced and misaligned factors, the initial alignment can still reduce the control efforts since control signals are subject to physical constraints with limited amplitude, like actuator saturation. It also helps to ensure that the vehicle parameters fall into the fully controllable range.

### Appendix A.2. Dynamic Model of the AUV

The details of dynamic model are presented in three subsections. The general approaches to building the model of a remotely operated vehicle (ROV), an AUV or other underwater vehicles are discussed in [21,22,23]. Some results are adopted, while further simplification of the modeling are derived to help design a more efficient control system.

• Actuator Dynamics

The dynamic relation of the setting value from the speed controller and real-time speed of motor is described in Equation (A1). A single lag model is used to model the real-time speed $\omega_{i}$ of the motor *i* and the setting input value $u_{i}$.

(A1) $$\begin{array}{c} \omega_{i}\left(s\right)=\frac{1}{T_{m}s+1}u_{i}\left(s\right). \end{array}$$

• Force and Moment Generation Process

This part analyses how the force and moment are generated and applied to the AUV. There are three terms applied on the vehicle, which are weight and buoyancy force and moment, water pump propulsion force and moment and AUV fluid dynamic force and moment.

*(a) Weight and Buoyancy*

The gravitational force and buoyant force in terms of body coordinate systems are,

(A2) $$\begin{array}{ccc} F_{w}^{b} & = & mg\left[\begin{array}{ccc} -\sin\theta & \cos\theta \sin\phi & \cos\theta \cos\phi \end{array}\right],\\ F_{B}^{b} & = & -\rho gV\left[\begin{array}{ccc} -\sin\theta & \cos\theta \sin\phi & \cos\theta \cos\phi \end{array}\right], \end{array}$$

where *g* is the gravitational acceleration, ρ is the fluid density and *V* is the volume of the fluid displaced by the vehicle. The moments generated by these forces can be expressed in terms of the positions of the center of mass *C* and the center of the buoyancy *B* [21].

(A3) $$\begin{array}{c} G_{W}=R_{C}\times F_{W},\;G_{B}=R_{B}\times F_{B}, \end{array}$$

where $R_{C}$ and $R_{B}$ are the respective positions of the center of mass and the center of buoyancy in the local coordinate system.

The AUV is aligned to be neutrally buoyant, which means $F_{w}=F_{B}$. It is also aligned to be naturally stable, which means the two of three Euler angles ϕ, θ are close to zero. The moment generated by the buoyancy force and gravity force can be simplified as follows,

(A4) $$\begin{array}{c} \tau_{xs}=F_{W}\cdot d\cdot \sin\left(\phi \right),\;\tau_{ys}=F_{W}\cdot d\cdot \sin\left(\theta \right), \end{array}$$

where *d* is the distance from the gravity center and buoyancy center. The more stabilization moments $\tau_{xs}$, $\tau_{ys}$ is always trying to maintain stability, which means the larger the *d*, the more stable the vehicle.

*(b) Water Pump Propulsion Force and Moment*

As discussed in Section 6.2.1, the resultant force $T_{b}$ and moments, $\tau_{y}$ and $\tau_{z}$, of the proposed propulsion system can be expressed as the vector sum of the force and moment form each individual pump in Equation (116).

*(c) AUV Fluid Dynamic Force and Moment*

The shape of the AUV is complex and therefore the modeling of its behavior is almost impractical. From the perspective of control, the simplified model at the operation points is desired.

The drag equation of fluid is

(A5) $$\begin{array}{c} f_{D}=\frac{1}{2}\rho v^{2}C_{d}A, \end{array}$$

where ρ is the density of the fluid, *v* is the speed of the object relative to the fluid, $C_{d}$ is the drag coefficient, *A* is the reference area. In the vector form,

(A6) $$\begin{array}{c} F_{D}=-\frac{1}{2}\rho \left[\begin{array}{ccc} C_{dx}A_{x} & 0 & 0\\ 0 & C_{dy}A_{y} & 0\\ 0 & 0 & C_{dz}A_{z} \end{array}\right]\left[\begin{array}{c} sign\left(v_{x}\right)v_{x}^{2}\\ sign\left(v_{y}\right)v_{y}^{2}\\ sign\left(v_{z}\right)v_{z}^{2} \end{array}\right]. \end{array}$$

Similarly, for the rotational moment, it is

(A7) $$\begin{array}{c} \tau_{D}=-\frac{1}{2}\left[\begin{array}{ccc} C_{\omega x} & 0 & 0\\ 0 & C_{\omega y} & 0\\ 0 & 0 & C_{\omega z} \end{array}\right]\left[\begin{array}{c} sign\left(\omega_{x}\right)\omega_{x}^{2}\\ sign\left(\omega_{y}\right)\omega_{y}^{2}\\ sign\left(\omega_{z}\right)\omega_{z}^{2} \end{array}\right]. \end{array}$$

To summarize, the overall force $f^{b}$ and moment $\eta^{b}$ applied to AUV are

(A8) $$\begin{array}{ccc} f^{b} & = & T_{b}+F_{D},\\ \eta^{b} & = & \tau_{G}+\eta +\tau_{D}=\left[\begin{array}{c} \tau_{xs}\\ \tau_{ys}\\ 0 \end{array}\right]+\left[\begin{array}{c} 0\\ \tau_{y}\\ \tau_{z} \end{array}\right]+\tau_{D}, \end{array}$$

where $T_{b}$, $F_{D}$, $\tau_{xs}$, $\tau_{ys}$, $\tau_{y}$, $\tau_{z}$ and $\tau_{D}$ are defined in Equations (116), (A4) and (A6).

• Rigid Dynamics of the AUV Body

Revisiting the notations in [24], the equations of motion for a rigid body subject to body force $f^{b}\in R^{3}$ and torque $\eta^{b}\in R^{3}$ applied at the center of mass and specified with respect to the body coordinate frame is given by the Newton–Euler equation in the body coordinate which can be written as,

(A9) $$\begin{array}{c} \left[\begin{array}{cc} mI & 0\\ 0 & J \end{array}\right]\left[\begin{array}{c} {\dot{v}}^{b}\\ \dot{\omega } \end{array}\right]+\left[\begin{array}{c} \omega \times mv^{b}\\ \omega \times J\omega \end{array}\right]=\left[\begin{array}{c} f^{b}\\ \eta^{b} \end{array}\right], \end{array}$$

where $v^{b}\in R^{3}$ is the body velocity, $\omega \in R^{3}$ is the body angular velocity, m∈R is the mass, $I\in R^{3\times 3}$ is an identity matrix and $J\in R^{3\times 3}$ is an inertial matrix. The effects of *added mass* [21] will influence the total mass *m* and total moment of inertial matrix *J*. There will be large uncertainties in those parameters as discussed in Equations (4) and (5).

The position and velocity of the AUV center of gravity are given by $p={\left[\begin{array}{ccc} x & y & z \end{array}\right]}^{T}$ and $v=\dot{p}\in R^{3}$, respectively, expressed to the spatial frame in North-East-Down orientation. Let R∈SO be the rotation matrix of the body axes relative to the spatial axes and vector. *R* can be parameterized by the ZYX Euler angles with ϕ, θ and ψ about the *x*, *y* and *z* axes, respectively.

(A10) $$\begin{array}{c} \begin{array}{c} R\left(\Omega \right)=\exp\left(\hat{z}\psi \right)\exp\left(\hat{y}\theta \right)\exp\left(\hat{x}\phi \right)\\ =\left[\begin{array}{ccc} c\theta c\psi & s\phi s\theta c\psi -c\phi s\psi & c\phi s\theta c\psi +s\theta s\psi \\ c\theta s\psi & s\phi s\theta c\psi +c\phi c\psi & c\phi s\theta s\psi -s\theta c\psi \\ -s\theta & s\phi c\theta & c\phi c\theta \end{array}\right] \end{array}, \end{array}$$

where $\hat{x}={\left[\begin{array}{ccc} 1 & 0 & 0 \end{array}\right]}^{T}$, $\hat{y}={\left[\begin{array}{ccc} 0 & 1 & 0 \end{array}\right]}^{T}$, $\hat{z}={\left[\begin{array}{ccc} 0 & 0 & 1 \end{array}\right]}^{T}$. By differentiating R(Ω) respect to time, we have the state equations of the Euler angles Ω,

(A11) $$\begin{array}{c} \dot{\Omega }=\Psi \left(\Omega \right)\omega , \end{array}$$

where Ψ(Ω) is give in Equation (2). In the ZYX Euler angle parameterization of rotation matrix, there are singularities at θ=±π/2. For the following discussion, we assume that the trajectory of AUV does not pass through the singularities. The motion equations of a rigid body are

(A12) $$\begin{array}{c} \begin{array}{c} \left[\begin{array}{c} \dot{p}\\ m\dot{v}\\ \dot{\Omega }\\ J\dot{\omega } \end{array}\right]=\left[\begin{array}{c} v\\ R^{T}\left(\Omega \right)f^{b}\\ \Psi (\Omega )\omega \\ \eta^{b}-\omega \times J\omega \end{array}\right] \end{array}. \end{array}$$

Equation (A12) summarizes the overall dynamic system of the AUV, where $f^{b}$ and $\eta^{b}$ are defined in Equation (A8).
